# A smartphone app to improve the safety of patients undergoing treatment with oral antineoplastic agents: 4 years of experience in a university hospital

**DOI:** 10.3389/fpubh.2022.978783

**Published:** 2022-11-04

**Authors:** Cristina Villanueva-Bueno, Roberto Collado-Borrell, Vicente Escudero-Vilaplana, José Luis Revuelta-Herrero, María Belén Marzal-Alfaro, Eva González-Haba, José Ángel Arranz-Arija, Santiago Osorio, Ana Herranz-Alonso, María Sanjurjo-Saez

**Affiliations:** ^1^Pharmacy Department, Hospital General Universitario Gregorio Marañón, Madrid, Spain; ^2^Instituto de Investigación Sanitaria Gregorio Marañón, Madrid, Spain; ^3^Medical Oncology Department, Hospital General Universitario Gregorio Marañón, Madrid, Spain; ^4^Hematology Department, Hospital General Universitario Gregorio Marañón, Madrid, Spain

**Keywords:** mhealth, cancer, oral antineoplastic agents, smartphone, safety

## Abstract

**Objective:**

This study aims to analyze the impact of the eOncosalud app on the management and follow-up of adverse effects (AE) in patients receiving oral antineoplastic agents.

**Material and methods:**

We performed an observational, prospective study of cancer outpatients treated with oral antineoplastic agents (OAA), monitored by the eOncosalud app between August 2017 and October 2021. Safety variables were collected from eOncosalud: the number of AE; severity of the AE according to CTCAE, version 4.03; timelapse from app installation to first recorded AE; automatic recommendations issued; and the patient's acceptance of the recommendations made. To assess the impact of the recommendations generated by the algorithm, we calculated the positive predictive value (PPV) as the number of recommendations accepted out of the total number of recommendations generated. Safety-related patient messages were also analyzed (AE, drug–drug interactions, drug administration).

**Result:**

The app was downloaded and used by 186 patients (58.0% women), with a mean age of 59.0 years. A total of 1,368 AE were recorded, the most frequent being fatigue (19.37%), diarrhea (18.20%), and skin changes (9.21%). Regarding the recommendations issued by the app algorithm, 102 patients received 344 information brochures, 39 patients received 51 recommendations for supportive care to control AE, 60 patients received 240 recommendations to visit their primary care doctor, 14 patients received 16 recommendations to contact their specialist pharmacist or oncologist-hematologist, and 34 patients received 73 recommendations to go to the emergency room. The suggestion to go to the emergency room and contact the specialist pharmacist or oncologist-hematologist had a PPV of 0.51 and 0.35, respectively. Half of the patients (50.4%) used the messaging module. A total of 1,668 messages were sent. Of these, 47.8% were related to treatment safety: AE, 22.7%; drug-drug interactions, 20.6%; drug administration, 3.6%; and missing a dose, 1.0%.

**Conclusions:**

The eOncosalud app enables close, real-time monitoring of patients treated with OAA. The automatic recommendations through the app's algorithm have optimized available healthcare resources. The app facilitated early detection of AE, thus enabling patients themselves to improve the safety of their treatment.

## Introduction

Safety is critical in patients undergoing treatment with oral antineoplastic agents (OAA). The adverse effects (AE) associated with antineoplastic agents affect patients' quality of life and impair the continuity of treatment, and, consequently, its effectiveness (1). Close follow-up of patients treated with OAA is essential to detect toxicity, facilitate early management, and reduce the severity and duration of AE (2, 3).

Various healthcare organizations consider information and communication technologies (ICT) an efficient approach for improving the care of cancer patients (4). In this sense, smartphone applications (apps) provide patients with greater autonomy and the possibility of communication and ensure that healthcare professionals receive information that will help them to improve patient follow-up and care (5–7). ICT tools can help manage AE remotely through algorithms capable of sending automatic responses, managing alerts if the parameters recorded are outside a healthy range, and enabling professionals to exchange messages with patients when they suspect a problem. Several studies have shown the added value of using mhealth in promoting adherence, empowering patients, increasing treatment safety through AE prevention, and improving quality of life in patients with chronic diseases such as diabetes, Parkinson's disease, cardiovascular disease, or cancer, which require continuous monitoring by healthcare professionals (8–12). Tabi et al. (13) analyzed the available mobile apps, focusing on those that help patients understand and take their medicines. The results showed that the majority of apps were developed by industry (73%, 11/15), and a minority of them were developed by healthcare professionals (15%, 3/20) or academia (2.1%; 7/328). The most frequent functions were reminder, symptom tracking, and the ability to share data with a family member or doctor. Another recent systematic review and meta-analysis of randomized controlled trials evaluated the efficacy of eHealth interventions on patient-reported outcomes during and after breast cancer treatment. The results showed that this type of tool is an acceptable and effective strategy to improve the quality of life, distress, self-efficacy, and fatigue in these patients (14).

A study conducted in the USA revealed more than 5,000 cancer-related apps and concluded that their use in clinical practice is increasing (15). However, the number of cancer-specific symptom-tracking apps remains limited. A recent systematic review of mobile health apps that could allow patients to record symptoms and patient-reported outcomes showed that only 27% were cancer-specific (16). In this regard, we developed and implemented a mobile app for cancer patients called eOncosalud (17, 18). This app, developed by health professionals, integrates relevant information about treatment with OAA and is focused on preventing and managing adverse effects through a proactive algorithm and real-time monitoring.

Our study aims to analyze the impact of eOncosalud on the management and follow-up of adverse effects in patients receiving OAA.

## Materials and methods

### Study design and setup

We performed an observational, prospective study of cancer outpatients treated with OAA and monitored using the app eOncosalud. The study was approved by the local ethics committee and conducted following the principles of the Declaration of Helsinki. Patients signed an informed consent document before entering the study.

### Study population

The study population comprised adult outpatients diagnosed with solid or hematological cancer who started treatment with targeted OAA between August 2017 and October 2021 in a university hospital. eOncosalud was available to all patients initiating treatment with OAA. The pharmacist proposed the app to patients at the beginning of the pharmaceutical care interview process. Along with the usual dispensing and information, the pharmacist taught patients how to use the app on their smartphone, registered them in the system, and gave them a user manual with technical specifications and practical examples of use.

We excluded patients who had received targeted OAA as part of a clinical trial, patients whose smartphones did not allow installation and use of the app, and patients who refused to participate.

### eOncosalud app

eOncosalud app has five modules:

- *An agenda or e-calendar of patient activity*: The agenda allows recording daily patient activity (appointments with healthcare professionals, medical tests, treatments…). The app collects all the activities or events (including the time of taking medication) that the patient has pending for the selected day. The backend of the platform calculates the pending events or activities, and returns a JavaScript Object Notation (JSON) message to the app, which is responsible for showing the user the pending events. As soon as the app receives the JSON message, it also calculates the necessary *push* notifications to notify the patient of the pending medication or event. When the patient resolves an event, it is saved via the Representational State Transfer application programming interface (REST API) to appear as completed in real-time.
- *A treatment record*: The user can include all their medications, doses, frequency, and times of administration in the app. When the day and time of the treatment record arrives, a *push* notification is sent to the devices to remind the user of the record. When the user confirms the treatment, a JSON message is sent via the REST API to save the exact time of confirmation. In this way, it is also marked as completed in the user's diary.
- *Self-monitoring of adverse effects module*: The adverse effects module is designed as a decision tree based on questions the app asks the user. Each question has possible answers associated with it, and in turn, these answers are associated with the next question. In this way, depending on the user's answer to a question, the next question may vary. This logic is obtained in the user login, and is stored so that the user experience is optimal. When the user reaches the end of the tree and saves his answers, the platform gives him feedback depending on the combination of questions and answers he has submitted.
- *Patient-pharmacist chat-messaging in real-time module*: The real-time messaging module enables communication between healthcare professionals and patients. When a healthcare professional sends a message to a patient, it appears as unread in the patient's inbox and a *push* notification is sent to the patient's device. It is stored in real-time through the REST API in the database. The time at which the user receiving the message reads it is also stored, so that it can be moved to the read mailbox and the traceability of the sending and reading of the message can be stored.
- *Education*: It contains useful information about diseases and treatment and links to related websites. The platform has a link to a website integrated with the app where informative and educational digital content is displayed. As it is a WordPress website, it is fully dynamic and content can be added and removed without the need to update the app or stop the service.

Additionally, eOncosalud provides a platform for healthcare professionals to monitor the information that patients record in the app. The professional platform has several privileges and partnerships to link healthcare professionals with their patients. When the healthcare professional logs in, the platform obtains the list of the patients. The healthcare professional can access the profile of all the patients associated with him/her. To manage security, in each call to the REST API, the token with the information of the user who is making the requests is added. This token checks the user's permissions. If an unprivileged user tries to make a restricted call, the API detects that the user does not have sufficient permissions to make the call and will return an error message.

**Figure 1:**
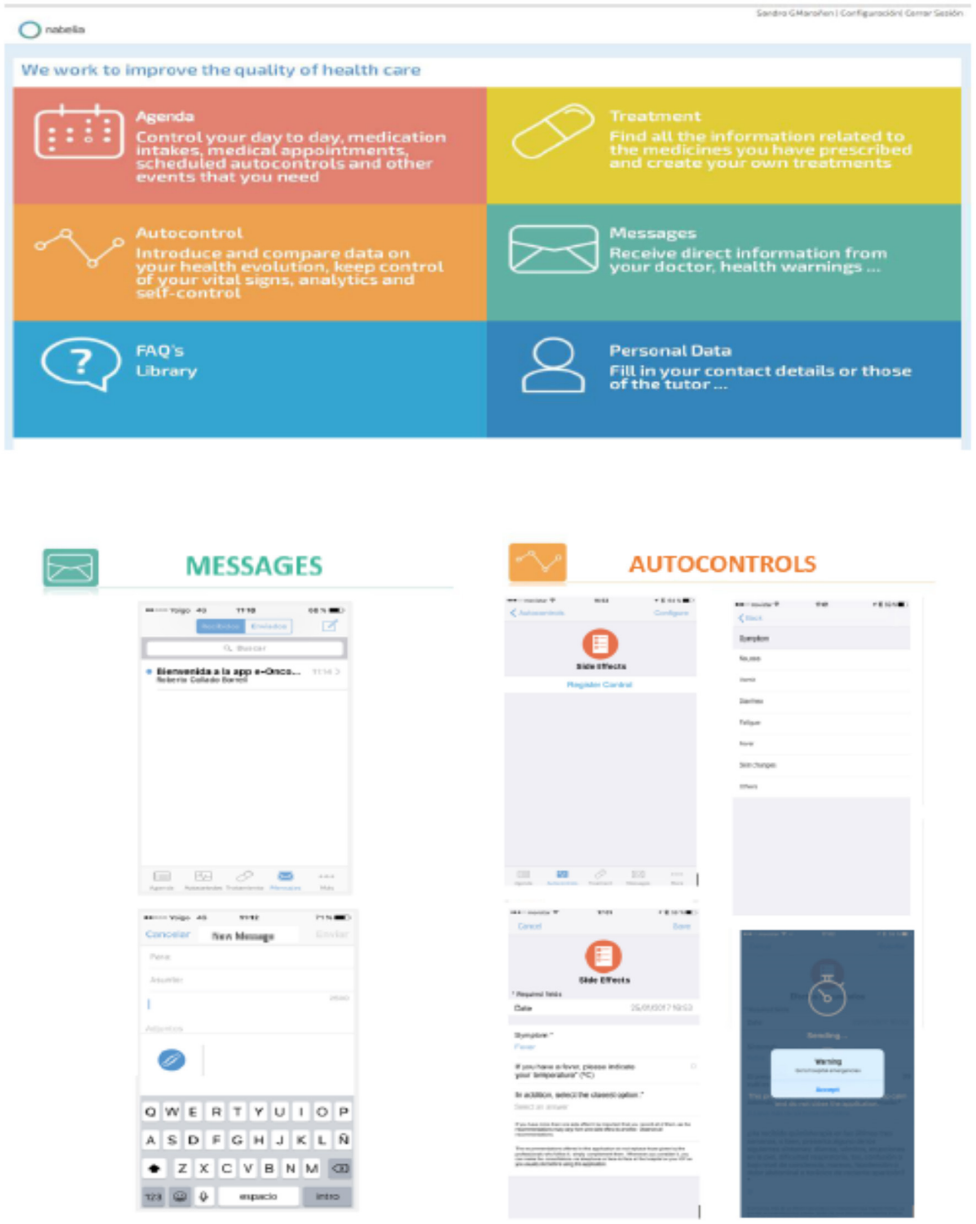
Functional diagram of the app eOncosalud.

The two modules involved in drug safety monitoring are described in detail below (Figure 1):

#### Self-monitoring of adverse effects module:

A multidisciplinary team of healthcare professionals composed of three pharmacists, a medical oncologist, and a hematologist at a university hospital in Spain was created to draft consensus recommendations for each of the most common AE observed in patients treated with OAA. The members were selected based on their extensive clinical and research experience in oncology-hematology and ICT management. The AE requiring recommendations were fatigue, diarrhea, nausea, vomiting, skin changes, fever, and others.

**Figure 2:**
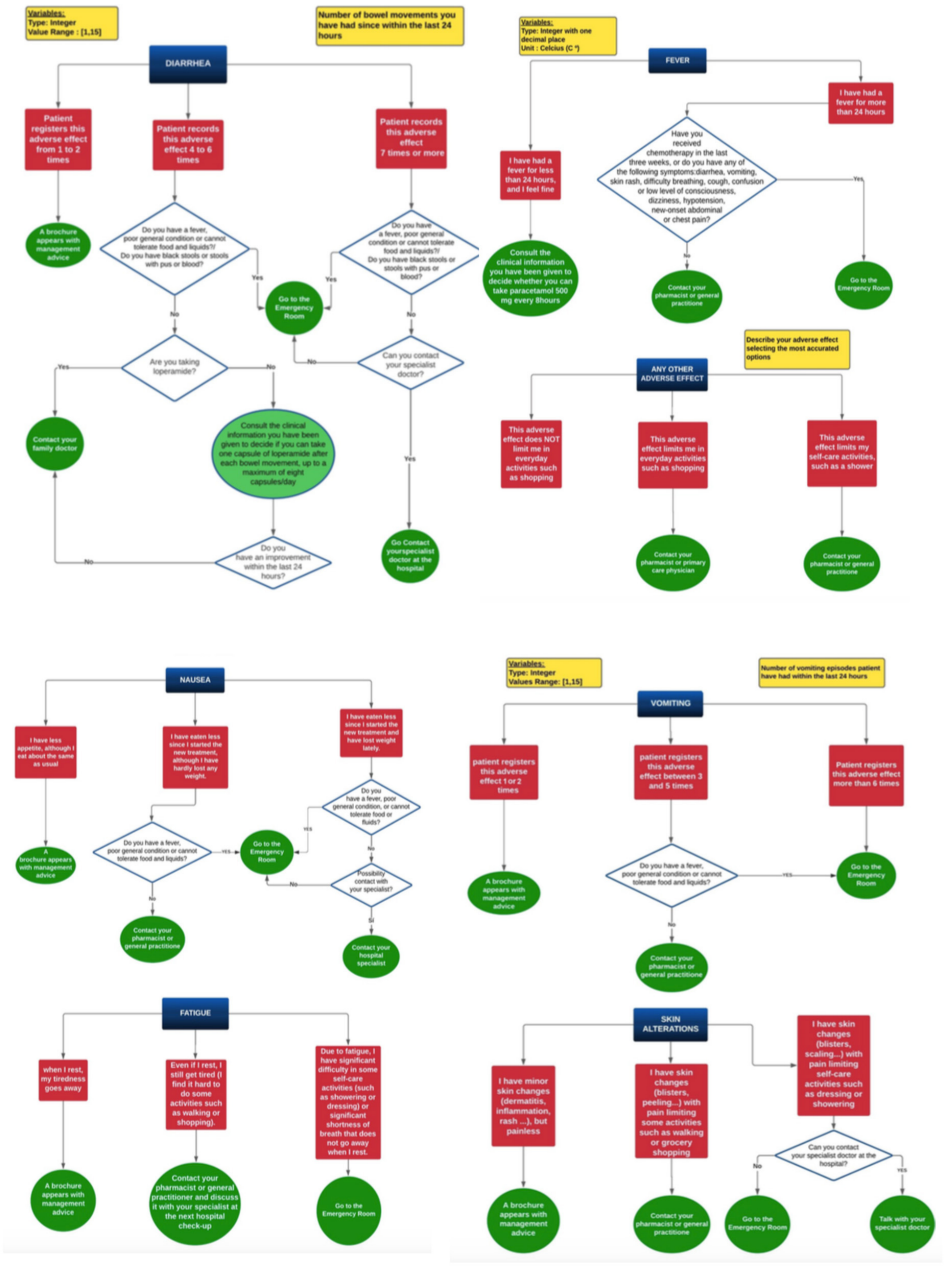
Algorithm to classify the AE by severity according to the CTCAE.

The algorithms are developed following a decision tree structure created by the team of healthcare professionals, depending on the patient's response, the platform continues to ask questions until it reaches the end of the tree, at which point the corresponding indication is given to the patient, depending on the message programmed in the tree for their set of answers. Thus, once the patient registers an AE, the system acts based on the algorithm to classify the AE by severity according to the CTCAE (grades 1–4) and provide expert group consensus recommendations on appropriate management (Figure 2).

The CTCAE was selected as a clinical-interpreted tool to allow an initial classification of the severity of AE and to help health professionals to manage the doses of the OAA according to technical data sheets. For grade 1 AE, general information brochures on the prevention and management of the selected AE are emitted. For the other grades, recommendations are given depending on the type and severity of the AE, ranging from advice on medication administration to messages for the patient to go to the emergency department. For mild AE management can be done in follow-up with the primary care physician, while moderate-severe AE requires specialist medical attention. In the case of diarrhea and vomiting, the algorithm considers the number of events registered in the previous 24 h, and when a predefined number of events is reached, various recommendations pop up. Other types of validated patient-reported outcomes tools, like NCI-PRO-CTCAE, can complement the information about AE. At the time of the app's development, the NCI-PRO-CTCAE was not validated.

#### Patient-pharmacist chat-messaging:

The app's messaging module functions as a private chat message that enables personal communication in real time between the patient and the pharmacist or pharmacists in charge. The platform is based on a REST API hosted in the cloud, with which both the patient app and the professional web interface communicate. The app and the web send and receive information through this API, which processes and manages the information, storing it in a relational database. This database stores all the information of the patients and the professionals, being able to obtain all the information of each patient in real time through the API, filtering it through the modules of the app. In addition to sending instantly individualized messages, eOncosalud can broadcast messages to a specific group of patients after scheduling a specific sending date. This feature makes it possible to emit text with no restrictions and attach relevant files. The messages are answered within 3 h of the question being asked between 8:00 am and 11:00 pm 7 days a week. The precautions taken to limit emergent problems reported through the app during the day and for serious problems outside office hours (23:00 to 8:00) were based on an initial Pharmaceutical Care consultation reinforcing the criteria for going to the emergency department (fever > 38°C, bleeding, etc.) and advising that the app is complementary to conventional methods of care and does not replace them. Besides, every time that a patient registers an AE or sends a message, the app automatically displays the warning signs for which they should go to the emergency department.

Patients use this module to express doubts about preventing or managing AE, potential drug-drug interactions, and drug administration.

### Data recorded and variables

The baseline parameters collected from the hospital's electronic medical records comprised demographic data (date of birth and sex), clinical data (tumor type), and pharmacotherapeutic data (specific OAA dispensed in the Outpatient Pharmacy Service). In addition, the mean time of use of the app was determined.

Safety variables were collected from eOncosalud, as follows: the number of AE recorded in the app; severity of the AE according to CTCAE version 4.03; timelapse from installation to first recorded AE; message sending time bands; automatic recommendations issued; and acceptance of the recommendation by the patient. Safety-related messages from the patient were analyzed (AE, drug-drug interactions, drug administration).

### Statistical analysis

Categorical variables were expressed as frequencies and percentages. Pearson's χ^2^ test and Fisher's exact test were used to study the association between categorical variables. The results are reported as mean and standard deviation for variables with a normal distribution and median and interquartile range for variables with a non-normal distribution.

To assess the impact of the recommendations generated by the algorithm, we calculated the positive predictive value (PPV) as the number of recommendations accepted out of the total number of recommendations generated. It measures all the patients who received a message to go to the emergency room or contact a specialist, who actually followed the recommendation.

The statistical analysis was carried out using SPSS Statistics for Windows, Version 21.0. Results with a *p* value less than 0.05 were considered statistically significant.

## Results

The app was offered to 389 patients. Of these, 47.8% (186/389) downloaded and used the app after signing the informed consent form. The median age was 59 (26–88) years and 58% were women. The average time of use of the app was 237 days. Breast cancer was the most frequent tumor, and palbociclib was the most frequent OAA. Table 1 describes the types of tumor and OAA among patients followed by eOncosalud.

**Table 1:** Type of tumors and OAA of the patient included in the study.

**Tumor**	**Number**	**Percentage**
**Breast** Palbociclib Ribociclib Abemaciclib Everolimus Capecitabine	42 19 10 5 7 1	22.6%
**Lung** Osimertinib Afatinib Gefitinib Erlotinib Crizotinib Alectinib Nintedanib	21 6 3 3 3 2 2 2	11.3%
**Prostate** Enzalutamide Abiraterone	18 10 8	9.7%
**Renal** Pazopanib Tivozanib Sunitinib Everolimus Cabozantinib	17 6 5 3 2 1	9.1%
**Ovarian** Olaparib Niraparib	16 10 6	8.6%
**Chronic lymphoid leukemia** Ibutinib Venetoclax	10 9 1	5.4%
**Gastrointestinal stromal tumor** Imatinib Regorafenib	9 7 2	4.8%
**Hepatocarcinoma** Sorafenib Regorafenib	9 6 3	4.8%
**Multiple myeloma** Lenalidomide Thalidomide	8 7 1	4.30%
**Sarcoma** Pazopanib Sunitinib Imatinib	7 4 2 1	3.8%
**Melanoma** Dabrafenib-trametinib Encorafenib-binimetinib	6 5 1	3.2%
**Acute myeloid leukemia** Sorafenib Dasatinib Imatinib	5 3 1 1	2.7%
**Chronic myeloid leukemia** Imatinib Dasatinib Nilotinib	4 2 1 1	2.1%
**Colonrectal** Capecitabina Encorafenib	4 2 2	2.1%
**Thyroid** Levantinib	2 2	1.6%
**Neuroendocrine tumor** Everolimus	2 2	1.1%
**Glioblastoma** Temozolamide	2 2	1.1%
**Myelodysplastic syndrome** Ruxolitinib	2 2	1.1%
**Gastric** Capecitabine	1 1	0.5%
**Pancreas** Olaparib	1 1	0.5%

OAA: Oral antineoplastic agents.

### Self-monitoring AE module:

The algorithm to manage the AE was used by 47.8% of patients (89/186). A total of 1,368 AE was recorded. The AE rate per patient was 7.35, and the time to onset of the first AE was 7 days (SD = 19.2) after the OAA started. During the first week of treatment, 25.8 % of patients had at least one AE. Table 2 shows the AE recorded by patients and their frequency and Table 3 classifies the AE by severity.

**Table 2:** The adverse effects and their frequency recorded by patients in the app.

**Adverse effect**	**Number of symptom recording events**	***N* (%)**
Fatigue	263	65 (19.25%)
Diarrhea	249	93 (18.23%)
Skin changes	126	46 (9.22%)
Nausea	118	40 (8.64%)
Vomiting	78	24 (5.71%)
Fever	39	22 (2.85%)
Others^*^	493	86 (36.09%)

* Other advert effects: pain (6.43%), swelling (5.48%), dizziness (5.12%), decreased appetite (5.11%), mucositis (2.56%), visual disturbance (1.83%), neuropathy (1.10%), alteration of blood pressure (0.73%), alopecia (0.51%), hives (0.37%), and insomnia (0.37%).

**Table 3:** Types of the adverse effects and their severity according to CTCAE clasification.

Severity^*^	**G1**		**G2**		**G3**		**G4**	
**Type of AE**	**No. AE**	**No. patients**	**No.AE**	**No. patients**	**No.AE**	**No. patients**	**No. AE**	**No. patients**
Fatigue	60	32	191	49	14	6	0	0
Diarrhea	106	66	77	58	43	14	23	3
Skin changes	110	38	11	8	5	4	0	0
Nausea	66	27	33	15	18	14	0	0
Vomiting	34	19	32	24	6	4	6	5
Fever	30	25	9	6	2	1	0	0
Others	266	76	197	28	30	12	0	0

AE, adverse effect;

* CTCAE, common terminology criteria for adverse events.

Regarding the recommendations issued by the app algorithm, 102 patients (54.8 %) received 344 information brochures, 39 patients (20.9%) received 51 recommendations for supportive care to control AE (paracetamol for pain, metoclopramide for nausea, and loperamide for diarrhea), 60 patients (32.4%) received 240 recommendations to visit their primary care doctor, 14 patients (7.5%) received 16 recommendations to contact their specialist pharmacist or oncologist-hematologist, and 34 patients (18.3%) received 73 recommendations to go to the emergency room. The suggestion to go to the emergency room and the suggestion to contact their specialist pharmacist or oncologist-hematologist had a PPV of 0.51 and 0.35, respectively.

Regarding treatment, 20 OAA (52.6%) were associated with AE requiring a visit to the emergency room. The OAA involved in this recommendation were as follows: palbociclib (4 patients), imatinib (3 patients), enzalutamide (3 patients), ibrutinib (3 patients), sorafenib (2 patients), abemaciclib (2 patients), crizotinib (2 patients), olaparib (2 patients), regorafenib (2 patients), and 1 each for the remainder (cabozantinib, capecitabine, dabrafenib, dasatinib, encorafenib, everolimus, lenvatinib, niraparib, osimertinib, pazopanib, and ribociclib). The AE presented by patients requiring emergency care were uncontrollable nausea with or without vomiting (46%), diarrhea (25%), and fever (17%).

### Patient-pharmacist chat-messaging:

Half of the patients (50.4%) used the messaging module. A total of 1,668 messages were sent (Table 4). Of these, 47.8% were related to treatment safety, as follows: AE, 22.7%; drug–drug interactions, 20.6%; drug administration, 3.6%; and missing a dose, 1.0%.

**Table 4:** The most frequent types of messages sent by patients through the app.

**Type of message**	**No. (1,668)**	**%**
Acknowledgment	395	23.68
Adverse effects	378	22.66
Drug interactions	343	20.56
Dispensing and return of medication	176	10.55
Drug home delivery	89	5.34
Problems, bugs, or suggestions about the app	80	4.80
Lifestyles or nutrition habits	67	4.02
Drug administration	60	3.60
Administration procedures	27	1.62
Forgetting to take medication	16	0.96
Preservation and/or transport of medication	5	0.30
Others	32	1.92

More than 95% of the messages were sent during business hours (58.3% from 8:00 to 15:59 and 38.1% from 16:00 to 23:59), only 3.6% outside the established hours (00:00–07:59).

Patients sent a mean of eight messages, and the mean of time until the first message was 15 days (SD = 19.4).

Beyond these figures that we have shown, the impact that the app is having due to the large number of messages of gratitude that we receive from patients, in which they highlight the peace of mind and confidence that having a pharmacy to contact offers them. As an example: “*I wanted to congratulate you on the app and thank you for the service provided through it. In my opinion, it has been very useful and helpful during the course of treatment, and I wish this type of app could be implemented in other oncology treatments*”.

## Discussion

In this study, we analyzed the impact of the eOncosalud app on the management of AE associated with OAA. Half of the patients reported at least one AE, and 26.0% of AE occurred during the first week of treatment. Thanks to eOncosalud, patients managed AE from home, thus enabling more efficient use of available healthcare resources.

International organizations such as the Institute for Safe Medication Practices (ISMP) (19) and the Joint Commission on Accreditation of Healthcare Organizations (JCAHO) consider that OAA are high-risk drugs (20). OAA can produce AE with severe consequences, although half are preventable. Nevertheless, AE are generally underreported by patients in clinical practice. Oakley et al. (21) observed that 23.0% of patients who had experienced an AE from an OAA had not reported it because they thought that it was not associated with the OAA or feared discontinuation of treatment. Therefore, it is essential to know the AE associated with each OAA to prevent or detect toxicity and ensure reasonable control of symptoms. In this sense, given their accessibility to the vast majority of the population and the possibility of remote monitoring, apps can fill this gap. However, few apps enable data to be recorded by the patient and monitored by the healthcare professional. eOncosalud contains a self-monitoring module that enables continuous registration of vital signs, patient measurements, and adverse effects (22). Table 5 shows some differences between similar apps available on the market and eOncosalud in terms of structure, development, and the possibility for patients to communicate with healthcare professionals.

**Table 5:** Differences between some of the apps available for cancer patients and eOncosalud.

**Name of the APP**	**Developers**	**To whom it is addressed**	**Objective**	**Modules**					**Type of interaction with healthcare professionals**	**Relevant differences to eOncoSalud app**
				**Calendar**	**Treatment**	**Symptoms/** **evolution**	**Education**	**Messaging**		
iCancerHealth	Mediociy, INC	Cancer patients	This app provides real-time monitoring and control of the patients.	x	x	x	x	x	Clinicians are advised to contact patients within 1 hour of receipt of a red alert. In the event of either amber or red alert, clinicians could access secure web pages to view the patients' symptom reports to assist in their clinical decision-making.	This app is similar in structure and development to eOncosalud. No information about an algorithm that issues recommendations according to the severity of recorded adverse effects. However, this app, like eOncoSalud, categorizes the adverse effect and the healthcare professional can prioritize the time to respond to the patient.
Becca - Breast Cancer support	Breast Cancer Now (Charity Association)	Breast cancer patients	This app provides support and inspiration to help patients to live well after breast cancer.			x	x		This app provides personalized cards focusing on topics ranging from personal experiences to upcoming cancer research.	No communication with healthcare professionals in real-time. No possibility to register adverse effects or treatments
BRIAN_The Brain tumor app	The brain tumor Charity.	Brain tumor patients	This app provides personalized cards focusing on topics ranging from personal experiences to upcoming cancer research.	x	x	x	x	x	Community chat rooms and answers to patients' questions from healthcare professionals.	No automatic and instantaneous recommendations are issued based on recorded adverse effects.
Cancer.net Mobile	ASCO (American Society of Clinical Oncology)	Cancer patients	This app mostly contains information pertaining to cancer.	x	x	x	x		System of annotations of doubts and questions to be asked at the next medical appointment.	Does not allow data export. No automatic and instant communication with healthcare professionals No automatic and instantaneous recommendations are issued based on recorded adverse effects.
CancerAid	CancerAid Pty Ltd	Cancer patients	This app is designed to help patients take control of physical and psychological adverse effects.		x	x	x	x	Interaction with healthcare professionals and other patients.	It does not allow data export. No automatic and instantaneous recommendations are issued based on recorded adverse effects.
Cancer Tracker	Health Stack LLC	Cancer patients	This app tracks symptoms and provides tips on symptom management.	x	x	x	x		System of annotations of doubts and questions to be asked at the next medical appointment.	No direct communication with healthcare professionals.
My Breast Cancer Advocate	Toliman	Cancer patients	This app provides general information and suggested questions to ask healthcare providers about breast cancer. It also has information related to breast cancer advocacy.		x	x	x	x	Connect with other patients who have faced or are facing the same decisions or issues. The app allows keep track and easy access to the doctors and their contact details. It is possible to contact the team via email or a call.	No automatic and instantaneous recommendations are issued based on recorded adverse effects.
Our Journey with Cancer	Phoenix Children's Hospital	Children cancer	This app helps families of children diagnosed with cancer identify what they need to know to safely care for their children at home after being discharged from the hospital.				x	x	Facilitate conversations between families and the healthcare teams.	No automatic and instantaneous recommendations are issued based on recorded adverse effects.
LivingWith Cancer support	Pfizer Inc.	Cancer patients	This app connects people to their clinicians in order to monitor conditions activity, episodes, and medication.	x	x	x	x		No contact with healthcare professionals via messaging	No direct communication with healthcare professionals. No automatic and instantaneous recommendations are issued based on recorded adverse effects.
Outcomes4Me Breast Cancer Care	Outcomes4me Inc.	Cancer patients	This app provides users with a plethora of personalized tools to help them manage their cancer.	x	x	x	x	x	The app has an Ask Center where patients can ask the team questions based on how they feel for support and guidance better management of their care.	No automatic and instantaneous recommendations are issued based on recorded adverse effects.
chemoWave For Cancer patients	Treatment Technologies & Insights	Cancer patients	This app helps patients track their activities and treatment experience, and provides them with information that enables them to work more effectively with their support team to better manage the adverse effects.	x	x	x			No communication with healthcare professionals or advice given by health professionals.	No direct courier service with healthcare professionals No automatic and instantaneous recommendations are issued based on recorded adverse effects.
Head & Neck Cancer Manager	@Point of Care	Head & Neck cancer patients	This app help patients track their progress and symptoms, manage their medication, and connect to healthcare providers.	x	x	x	x	x	Connect to care providers so they can monitor patient progress between visits.	No automatic and instantaneous recommendations are issued based on recorded adverse effects.
The-Optimal-Lymph-Flow health IT system (TOLF)	Rutgers University	Breast cancer survivors	This app is a patient-centered, web-and-mobile-based educational and behavioral mHealth interventions focusing on self-care strategies for lymphedema symptom management.		x	x	x		No instant contact with healthcare professionals. The app has a daily 5-min routine avatar simulation video of lymphatic exercises providing a unique way of helping breast cancer survivors to establish their own self-care routine by following the video and educational material.	No direct communication with healthcare professionals.

Own elaboration based on data extracted from each mobile app website and from the app store and google play websites.

In our study, 47.8% of patients used the self-monitoring module to record an AE, and more than a quarter of the patients reported them during the first week of treatment. Weaver et al. (23) analyzed safety in 26 patients with breast and colorectal cancer treated with capecitabine and observed that patients could safely optimize their dose via an app by recording AE from home. In a clinical trial conducted in patients with lung cancer, Carlson et al. (24) demonstrated that early detection of psychosocial disturbances could help healthcare professionals to identify patients at greater risk for these disorders and intervene to prevent the development of crises, especially in patients with greater symptom burden.

In a study that analyzed the role of the app “*Advanced Symptom Management System*” for in-home monitoring of chemotherapy-induced symptoms such as nausea, vomiting, mucositis, diarrhea, and hand-foot syndrome, it was observed that patients felt confident with real-time monitoring and that they were effectively participating in their care management (25). Another study of 355 breast cancer patients with lymphedema showed that app-based interventions significantly improved pain, tenderness, number of lymphedema symptoms, and distress over symptoms (26). Other apps, such as iCancerHealth (27), can record measures such as blood glucose, diet, and oxygen associated with AE. However, compared with the many apps available worldwide, eOncosalud provides a proactive algorithm that enables patients to control symptoms at home. Depending on the type and severity of the AE recorded by the patient, the app issues personalized recommendations instantaneously. Lu et al. (16) performed of mobile health apps with symptom trackers. The majority of apps allowed for the inclusion of additional symptoms (80%), symptom severity rating (95%), ability to annotate notes to symptoms (76%), and graphical depiction of symptoms over time (61%), such as eOncosalud. However, for apps that included symptom severity scales, Common Terminology Criteria for Adverse Events were not detailed and ratings reflected a patient's subjective symptom severity.

ICT, specifically mobile device-based platforms, have positively impacted health outcomes. These tools have improved the management of various diseases, with results showing a remarkable decrease in complications and hospital admissions (28). Ortiz et al. (29) concluded that the use of text messaging by patients with diabetes led to a significant decrease in HbA1c levels, improved medication adherence, and reduced emergency room visits. Another study evaluated the effect of remote monitoring in patients with heart failure, which significantly improved patient health outcomes (30). Although we did not use a control group to demonstrate that our app improves resource consumption, it did enable the frequency of emergency room visits to be reduced and patients to be referred to their primary care physician for management of AE identified with the app.

A recent systematic review of cancer patient empowerment apps (31). describes and evaluates the features, quality, and evidence supporting these apps. App contents were very varied, and focused on communication, social support, and cancer treatment information. The app readability was excluded for the average reader. In this regard, eOncosalud has been designed by a multidisciplinary team of healthcare professionals and it has been rigorously tested to ensure that the app is usable and adaptable for diverse groups of cancer survivors. In addition, the collaboration between oncologists, hematologists, pharmacists, and app developers has optimized e-Oncosalud to enhance PRO assessment and increase symptom recognition and enhance patient-health professional communication. eOncosalud is one of the few cancer apps available on the market that has been awarded with the AppSaludable Quality Seal (32).

### Future development

The next steps in the development of this app are, first, the incorporation of PRO-CTCAE in a complementary way to the CTCAE already present in the algorithm as well as encouraging the completion of adherence, quality of life, and satisfaction tests available in the app.

Secondly, the development of our own indicators to evaluate the impact of the app on improving the quality of care and patient monitoring.

Finally, we are working hard to integrate automatically the information in the app into the patient's medical record.

### Limitations

It should be noted that, despite the study's prospective design and large sample, the absence of randomization could detract from the robustness of the results as well as the use of a comparator control group. Secondly, we do not know the number of apps containing an algorithm for managing AE associated with chemotherapy, with little evidence on improving patient safety. Finally, we did not analyze some variables, such as reduced visits to the emergency room, patient empowerment, or usability to better evidence the added value of the app for the patient. Also, knowing why patients declined to use the app would have provided us with more information on the degree of acceptance.

### Conclusions

The eOncosalud app enables close, real-time monitoring of patients treated with OAA. The automatic recommendations through the algorithm optimized available healthcare resources. eOncosalud facilitated the early detection of AE, thus enabling patients themselves to improve the safety of their treatment.

## Data availability statement

The raw data supporting the conclusions of this article will be made available by the authors, without undue reservation.

## Ethics statement

The studies involving human participants were reviewed and approved by EONC-APP-2018. The patients/participants provided their written informed consent to participate in this study.

## Author contributions

VE-V and RC-B had an equal contribution to the study, VE-V, RC-B, and CV-B wrote the first draft and defined the research question and objectives. VE-V, RC-B, JR-H, JA-A, SO, and AH-A carried out the technical development of the app. VE-V, CV-B, RC-B, MM-A, JR-H, and EG-H included the patients and followed them up. VE-V, RC-B, AH-A, and MS-S were responsible for the research activity plan and its execution. All authors reviewed and approved the final manuscript.

## Funding

This project was supported through funding received from FEDER Project PI13/02056. Support was co-financed by the European Regional Development Fund (FEDER)-A way of making Europe.

## Conflict of interest

The authors declare that the research was conducted in the absence of any commercial or financial relationships that could be construed as a potential conflict of interest.

## Publisher's note

All claims expressed in this article are solely those of the authors and do not necessarily represent those of their affiliated organizations, or those of the publisher, the editors and the reviewers. Any product that may be evaluated in this article, or claim that may be made by its manufacturer, is not guaranteed or endorsed by the publisher.

## Abbreviations

AE: adverse effects
App: mobile application
CTCAE: common terminology criteria for adverse events
ECOG: eastern cooperative oncology group
ICT: information and communication technologies
OAA: oral antineoplastic agents
PPV: positive predictive value.
